# Brain dynamics of attentional, default-mode and limbic networks are disrupted at rest in post-COVID-19 syndrome

**DOI:** 10.1016/j.bbih.2026.101274

**Published:** 2026-05-25

**Authors:** Marie-Stephanie Cahart, Owen O’ Daly, Ziyuan Cai, Nicole Mariani, Alessandra Borsini, Valeria Mondelli, Brandi Eiff, Silvia Rota, Timothy Nicholson, Laila Rida, Adam Hampshire, Ottavia Dipasquale, Lia Fernandes, Federico Turkheimer, Steven C.R. Williams, Daniel Martins

**Affiliations:** a Department of Neuroimaging, Institute of Psychiatry, Psychology and Neuroscience, King's College London, UK; b Department of Psychological Medicine, Institute of Psychiatry, Psychology and Neuroscience, King's College London, London, UK; c Department of Psychosis Studies, Institute of Psychiatry, Psychology and Neuroscience, King's College London, London, UK; d Institute for Human and Synthetic Minds, King's College London, UK; e Department of Clinical Neursciences and Mental Health, Faculty of Medicine, University of Porto, Porto, Portugal; f RISE-HEALTH (Neurosciences thematic line), Department of Clinical Neursciences and Mental Health, Faculty of Medicine, University of Porto, Porto, Portugal

**Keywords:** Post-COVID-19 syndrome, Resting-state fMRI, Dynamic functional connectivity, LEiDA, Brain states, Attention, Limbic system, Default mode network, Neuroinflammation

## Abstract

**Background:**

Post-COVID-19 Syndrome (PCS) is characterised by persistent fatigue, cognitive impairments, and affective symptoms, yet its underlying neural mechanisms remain poorly understood. While static neuroimaging studies have identified resting-state connectivity abnormalities in PCS, such approaches fail to capture the brain's dynamic functional organisation. This represents a missed opportunity to understand how alterations in large-scale network interactions may contribute to the fluctuating symptom profile of PCS. Cognitive and emotional processes rely on the brain's capacity to flexibly reconfigure large-scale networks over time; disruptions in this dynamic repertoire may therefore play a role in PCS pathophysiology.

**Methods:**

Resting-state fMRI data were acquired from 20 individuals with PCS (mean age = 41.8 years, SD = 9.4) and 20 age- and sex-matched healthy controls (mean age = 40.6 years, SD = 8.1) using a multi-echo sequence. Following denoising with multi-echo independent component analysis, we applied Leading Eigenvector Dynamics Analysis (LEiDA) to identify recurrent patterns of whole-brain phase synchrony. The optimal number of dynamic brain states was determined using the Dunn index. For each state, we quantified probability of occurrence, lifetime, and transition probabilities, and mapped spatial topographies onto canonical functional networks. Group differences were assessed using ANCOVAs controlling for age, sex, and handedness. Exploratory associations with clinical symptoms, cognitive performance, and inflammatory markers were examined using both frequentist and Bayesian approaches.

**Results:**

Five recurrent dynamic brain states were identified. Compared with controls, PCS participants showed reduced probability of occurrence and shorter lifetime of a visual/dorsal attention state, alongside increased probability of a limbic/default mode network (DMN) state. PCS was also characterised by tentative reduced transitions between visual/dorsal attention and frontoparietal–DMN states, and increased transitions from somatomotor/visual states toward the limbic-DMN configuration. Exploratory analyses (uncorrected for multiple comparisons) suggested that greater expression of the limbic-DMN state was associated with lower global cognitive performance (MoCA) and higher serum IL-1β levels, although these associations did not survive correction for multiple comparisons.

**Conclusions:**

PCS is associated with a reorganisation of intrinsic brain dynamics, marked by a shift from externally oriented attentional states toward limbic-DMN configurations and reduced transition flexibility. These findings suggest that PCS may involve alterations in the dynamic balance of large-scale brain systems supporting attention and internally oriented processing. While exploratory, the observed patterns are consistent with a potential link between brain-state dynamics, cognitive function, and inflammatory signalling, and provide a systems-level framework for future studies of post-viral brain dysfunction.

## Highlights

• Post-COVID-19 Syndrome (PCS) is linked to altered intrinsic brain dynamics at rest.
• Patients show reduced engagement of visual and attentional brain states.
• Increased recruitment of limbic-DMN states suggests a tentative shift toward more internally charged brain activity.
• Brain-state dynamics are exploratorily associated with global cognition and IL-1β.
• Reduced brain-state flexibility may underlie cognitive and emotional symptoms in PCS.

## 1 Introduction

Post-COVID-19 Syndrome (PCS), also referred to as Long COVID, affects a considerable number of individuals after infection with SARS-CoV-2 (Davis et al., 2021; Nalbandian et al., 2021). Even months after the acute phase has resolved, many people continue to experience symptoms such as fatigue, poor concentration, slowed thinking, mood changes, and issues with memory (Davis et al., 2021; Nalbandian et al., 2021; Cipolli et al., 2023). These difficulties occur even in individuals who were not hospitalised and may persist despite normal findings on conventional brain imaging. The underlying causes of PCS are still being investigated, but current evidence suggests that the brain may be particularly vulnerable to subtle changes in how it functions and our different brain regions communicate with each other, especially in the context of inflammation (Nalbandian et al., 2021; Proal and VanElzakker, 2021; Peluso and Deeks, 2024).

Resting-state functional MRI (rs-fMRI) has become a widely used tool for investigating large-scale brain network organisation in both health and disease (Biswal and Uddin, 2025). By measuring spontaneous fluctuations in the Blood Oxygenation Level Dependent (BOLD) signal while participants are not engaged in any specific task, rs-fMRI reveals patterns of synchronised activity across different brain regions, thus uncovering the brain's intrinsic functional architecture. These networks - such as the default mode, frontoparietal, limbic, and attention systems - are involved in diverse domains including emotion regulation, memory, self-reflection, and goal-directed cognition (Biswal and Uddin, 2025). Notably, rs-fMRI measures have been shown to predict individual patterns of brain engagement during cognitive and affective tasks, offering insight into latent network dynamics that shape behaviour even at rest (Biswal and Uddin, 2025; Kocak, 2021). Several studies using rs-fMRI have shown that PCS is associated with changes in connectivity within and between key large-scale brain networks (Churchill et al., 2023; Zhao et al., 2024; Kesler et al., 2024; Troll et al., 2025; Chen et al., 2025; Samanci et al., 2025; Zhang et al., 2025; Wingrove et al., 2023; Muccioli et al., 2023; Dacosta-Aguayo et al., 2024). In particular, altered connectivity has been observed in the default mode network (DMN), the salience network, and frontoparietal systems, which are responsible for executive control and attention^18, 19 20^. These abnormalities often correlate with symptoms like fatigue and mental fog (Zhang et al., 2025). However, most of these studies have used “static” analyses that summarise brain functional connectivity as if it remained the same across the entire scan (Menon and Krishnamurthy, 2019). In reality, brain connectivity is constantly evolving (Hancock et al., 2025). Even at rest, the brain shifts from one functional state to another over the course of seconds. These states, which represent groups of brain regions whose activity is highly synchronised at a given moment in time, reflect distinct modes of neural processing (Vohryzek et al., 2020). The way these states emerge and take turns activating across time offers insights into the brain's moment-to-moment responsiveness and its ability to flexibly adapt to changing demands (Hancock et al., 2025; Kurtin et al., 2023; Cabral et al., 2017).

In healthy individuals, the brain naturally moves between states related to attention, sensory awareness, emotion, and self-monitoring (Vohryzek et al., 2020). These states are temporary, and the brain usually does not get “stuck” in any one of them (Hancock et al., 2025). In psychiatric and neurological conditions such as depression, schizophrenia or body dysmorphic disorder, this balance is often disrupted. For example, some individuals spend more time in states related to internal thinking or negative emotion and explore fewer states related to attention and engagement with the external world (Alonso Martinez et al., 2020; Alonso et al., 2023; Cahart et al., 2025; Hancock et al., 2023; Wong et al., 2021). Because individuals with PCS share many of these symptoms, it is reasonable to suspect that something similar may be happening in their brains. One method that allows us to investigate this in detail is called Leading Eigenvector Dynamics Analysis (LEiDA)(Vohryzek et al., 2020; Cabral et al., 2014). This technique examines moment-to-moment changes in how different brain regions synchronise their activity. LEiDA identifies a set of distinct brain states and shows how frequently each state occurs, how long it lasts, and how often the brain switches between them. Although LEiDA has not yet been widely used in PCS research, early connectivity studies suggest that individuals with PCS may show reduced network efficiency over time (Zhang et al., 2025), which in turn provides a potential backbone for less flexible brain dynamics.

In this context, we set out to use LEiDA to explore how large-scale brain states unfold over time in individuals with PCS during resting-state fMRI. Building on prior research linking PCS to cognitive and affective dysfunction (Hampshire et al., 2021, 2024) - as well as emerging evidence of altered network connectivity in fatigue-related and inflammatory conditions (Goldsmith et al., 2023) - we formulated three core hypotheses. First, we hypothesised that individuals with PCS would spend more time in brain states dominated by the DMN and limbic regions. These areas are associated with internally directed cognition, self-referential thought, and affective processing, which may become predominant in the context of fatigue, emotional dysregulation, and withdrawal from external demands (Whitfield-Gabrieli and Ford, 2012). Second, we expected reduced occupancy of brain states engaging frontoparietal and dorsal attention networks, which are critical for sustained attention, goal-directed behaviour, and environmental monitoring (Vossel et al., 2014; Seeley et al., 2007). A diminished ability to access or maintain these externally focused states could help explain the common experiences of mental fog, distractibility, and slowed cognitive processing reported by PCS patients. Third, we hypothesised that individual differences in brain-state dynamics - specifically, reduced engagement of attention-related states and increased expression of DMN-limbic configurations - would be associated with clinical symptom severity, cognitive performance deficits (particularly in attention), and elevated peripheral markers of inflammation. Together, these hypotheses aimed to link brain dynamics with the neurocognitive and immunological signatures of PCS.

## 2 Methods

### 2.1 Study design and participants

As described elsewhere, this study employed a single-site observational case-control design. All participants, both PCS cases and recovered controls, were required to have had biologically confirmed SARS-CoV-2 infection at least three months prior to enrolment, consistent with the WHO clinical case definition of Post-COVID-19 condition (Soriano et al., 2022). Individuals in the PCS group were characterised by persistent symptoms lasting ≥3 months after infection, whereas recovered controls were required to be fully asymptomatic, with complete resolution of all post-COVID symptoms. We included individuals with mild acute COVID-19 only, to minimize potential confounding effects of hospitalisation, hypoxia, or severe systemic inflammation on brain perfusion and metabolism, and to ensure that any observed group differences were not attributable to the neurological sequelae of moderate–severe disease. All participants were required to have had biologically confirmed SARS-CoV-2 infection prior to enrolment. Confirmation was based on either a positive reverse-transcription polymerase chain reaction (RT-PCR) test from a nasopharyngeal swab or documented serological evidence of prior infection, in accordance with UK national testing guidelines at the time of acute illness. Participants with only clinically suspected but biologically unconfirmed COVID-19 were not included. Recovered healthy control participants were required to have a history of biologically confirmed SARS-CoV-2 infection, followed by complete resolution of all acute and post-acute symptoms. To minimize the risk of including individuals with subtle or subclinical post-COVID manifestations, control participants were screened using the same standardized clinical instruments applied to the PCS group, including the Fatigue Assessment Inventory. Only individuals scoring below established clinical thresholds, and reporting no persistent fatigue, cognitive complaints, affective symptoms, autonomic dysfunction, or post-exertional malaise at the time of assessment, were included as healthy controls. In addition, control participants underwent the same cognitive assessment battery and clinical interview as PCS participants, and individuals demonstrating clinically relevant deviations from normative performance or reporting residual symptoms were excluded. This stringent screening strategy ensured that the control group represented a recovered post-COVID population without ongoing symptomatology, rather than asymptomatic or mildly affected PCS cases.

We recruited participants through King's College Hospital NHS Trust and surrounding community networks. Eligible individuals were aged 16–65 years, fluent in English, and capable of providing informed consent. We excluded individuals with a history of major neurological or psychiatric disorders, current substance misuse, systemic or central nervous system inflammatory conditions, chronic respiratory or cardiac disease, recent vaccination (within two weeks), pregnancy or breastfeeding, BMI >30, or current use of immunomodulatory or psychoactive medications. We used the global severity score of Fatigue Assessment Inventory (FAI) to pre-screen participants for symptom severity (Schwartz et al., 1993). Individuals scoring greater than 4 on the global fatigue severity scale were classified into the PCS group with persistent fatigue, while those scoring 4 or below were included as recovered controls. The threshold of >4 was selected to ensure inclusion of individuals with clinically meaningful levels of fatigue, consistent with prior applications of the FAI in post-viral and chronic fatigue contexts (Schwartz et al., 1993). Groups were balanced for age, sex, BMI, and acute COVID-19 severity. To specifically isolate mechanisms associated with symptom persistence following SARS-CoV-2 infection, we selected a control group consisting of individuals with biologically confirmed infection who fully recovered and were rigorously screened to exclude any residual symptoms. This design allows for comparison between divergent clinical trajectories following infection (persistent vs resolved), rather than between infected and infection-naïve individuals. The study received ethical approval from the UK Health Research Authority (IRAS ID: 308661) and was conducted in accordance with the Declaration of Helsinki and Good Clinical Practice (GCP) guidelines. All participants provided written informed consent prior to study procedures.

We conducted an a priori power analysis using G*Power (version 3.1) to estimate the required sample size for detecting group differences in dynamic functional connectivity metrics using analysis of covariance (ANCOVA). Assuming a medium to large effect size (Cohen's f = 0.30, equivalent to ηp^2^≈0.08), alpha = 0.05, power (1 − β) = 0.80, and three covariates (age, sex, and handedness), the estimated sample size required per group was 19. Our sample of 20 participants per group therefore provides adequate power to detect effects in this range. Given the modest sample size, the study is likely underpowered to detect small-to-moderate effects. Accordingly, our analytical approach prioritised estimation over dichotomous inference, with emphasis placed on effect sizes, confidence intervals, and cross-modal consistency. Observed effects should therefore be interpreted as preliminary and most informative for medium-to-large effect sizes.”

### 2.2 Procedures

Each participant completed a single study visit that included clinical assessments, venous blood collection, and a multimodal MRI scan. Clinical assessments included medical and psychiatric history, physical examination, vital signs, and a structured battery of standardized questionnaires. Fatigue was assessed using both the full Fatigue Assessment Inventory (Schwartz et al., 1993) and the Multidimensional Fatigue Inventory (Smets et al., 1995) to differentiate between mental and physical fatigue. Symptoms of autonomic dysfunction were evaluated using the Composite Autonomic Symptom Score (COMPASS-31) (Sletten et al., 2012), while mood and anxiety were measured using the Hospital Anxiety and Depression Scale (HADS)(Zigmond and Snaith, 1983). Sleep quality was assessed with the Pittsburgh Sleep Quality Index (Buysse et al., 1989), and respiratory symptoms were rated using the MRC Dyspnoea Scale (Bestall et al., 1999). Additional measures included the DePaul Symptom Questionnaire (Sunnquist et al., 2019), the American College of Rheumatology (ACR) fibromyalgia criteria (Wolfe et al., 2016), the PAIN Detect questionnaire (Shaygan et al., 2013), and Montreal Cognitive Assessment (Nasreddine et al., 2005). Most self-report questionnaires were completed remotely via the Qualtrics platform within 72 h of the in-person visit. All participants underwent a structured diagnostic assessment using the Mini International Neuropsychiatric Interview (MINI) to exclude current or past major psychiatric disorders, including mood, anxiety, psychotic, and substance use disorders.

### 2.3 Blood analysis

Venous blood samples (up to 30 mL) were collected on the day of imaging. Standard hematological and biochemical panels - including erythrocyte sedimentation rate (ESR), C-reactive protein (CRP), thyroid-stimulating hormone (TSH), free thyroxine (T4), and liver function tests - were processed by Synnovis (Viapath) at King's College Hospital NHS Foundation Trust. Serum samples were frozen at −80 °C and later assayed for cytokines and glial markers using electrochemiluminescence immunoassays (Meso Scale Discovery, Rockville, MD, USA). The cytokine panel included interleukin-1 beta (IL-1β), interleukin-2 (IL-2), interleukin-4 (IL-4), interleukin-6 (IL-6), interleukin-8 (IL-8), interleukin-10 (IL-10), interleukin-12p70 (IL-12p70), interleukin-13 (IL-13), tumor necrosis factor-alpha (TNF-α), and interferon-gamma (IFN-γ). Glial markers included glial fibrillary acidic protein (GFAP), measured with the ultrasensitive kit, and S100β. Assays were performed in duplicate. Values below the detection limit were discarded. This led us to discard all quantifications of IL-2, IL-4 and IL-12p70. Outliers greater than three standard deviations from the group mean were winsorized. No analyte had more than 10% missing data, therefore all data were included.

### 2.4 Cognitron

Cognitive performance was assessed online using a battery of computerized tasks from the Cognitron platform, covering delayed verbal memory, working memory, executive function, attention, motor coordination, and spatial manipulation (Hampshire et al., 2024). Tasks included Verbal Analogies (abstract reasoning and semantic integration), Immediate and Delayed Prospective Memory (episodic memory and planning), 2D Spatial Manipulation (visuospatial working memory and mental rotation), Motor Control (sensorimotor coordination and reaction consistency), Spotter (vigilance and sustained attention), and Block Reasoning (fluid intelligence and rule-based problem solving). Each task involves trial-level response collection, capturing both accuracy and reaction time. Performance metrics were transformed into standardized deviation-from-expected (DFE) scores using normative models derived from a large independent sample (n > 20,000), adjusted for age, sex, handedness, and harmonized ethnicity. DFE scores represent standardized residuals (observed minus expected performance divided by normative standard deviation) and were computed separately for accuracy and reaction time.

## 3 Neuroimaging data acquisition and processing

### 3.1 MRI acquisition

We acquired MRI data using a 3T GE Healthcare MR750 scanner (GE Medical Systems, Milwaukee, WI), equipped with a 32-channel receive-only head coil. The imaging protocol was part of the larger FALCS neuroimaging study and included high-resolution anatomical scans, quantitative BOLD sequences, and cerebral perfusion imaging. For the purposes of this study, we focused exclusively on resting-state functional MRI (rs-fMRI) data to assess dynamic brain connectivity.

T1-weighted structural images were acquired using a 3D magnetisation-prepared rapid acquisition gradient echo (MPRAGE) sequence with the following parameters: repetition time (TR) = 2300 ms, echo time (TE) = 2.98 ms, inversion time (TI) = 900 ms, and voxel size = 1 mm^3^ isotropic. These anatomical images served as the reference for spatial registration and normalisation.

Resting-state fMRI data were acquired using a multi-echo echo-planar imaging (ME-EPI) sequence with full k-space sampling at each echo. Three echoes were collected per volume with echo times (TEs) of 13 ms, 28 ms, and 44 ms. The repetition time (TR) was 2000 ms, and a total of 240 vol were acquired over 8 min, following four initial dummy scans to allow for signal stabilization. Imaging covered the whole brain with 32 near-axial slices oriented along the anterior commissure–posterior commissure (AC–PC) line, each 3 mm thick with a 1 mm inter-slice gap. The field of view (FOV) was 240 mm, and the flip angle was set to 80°. During scanning, participants were instructed to remain still, keep their eyes open, and fixate on a central crosshair.

### 3.2 Preprocessing

We conducted preprocessing using FSL (version 6.0) and tedana.py (version 0.0.10; https://tedana.readthedocs.io/en/latest). The first step involved registering the anatomical MPRAGE image to Montreal Neurological Institute (MNI) space. Functional volumes from the first echo of each rs-fMRI time series were aligned to the first volume using mcflirt in FSL. The resulting transformation matrices were then applied to the remaining echoes, ensuring consistent alignment across all echoes. We then performed T2*-weighted optimal combination of the echoes to enhance BOLD contrast, and denoised the data using multi-echo independent component analysis (ME-ICA) implemented in tedana.py (Kundu et al., 2012, 2013). This method decomposes the data into independent components and classifies them as BOLD or non-BOLD based on their TE-dependence. Components identified as non-BOLD - typically reflecting motion, physiological noise, or scanner-related artefacts - were regressed out of the optimally combined data, yielding a denoised multi-echo BOLD dataset. The denoised time series were then registered to the corresponding MPRAGE structural image using epi_reg and subsequently normalised to MNI space using the previously computed anatomical transformations. All functional images were resampled to 2 mm^3^ isotropic resolution. To enhance spatial comparability and reduce high-frequency noise, the data were smoothed using a Gaussian kernel with 6 mm full-width at half-maximum (FWHM). We applied high-pass temporal filtering with a cut-off frequency of 0.01 Hz to remove low-frequency drifts and scanner instabilities. Finally, to account for residual non-neuronal sources of variance, we regressed out the average signal from white matter (WM) and cerebrospinal fluid (CSF). WM and CSF masks were defined in MNI space using the MNI152 standard template and applied to each participant's preprocessed data. The extracted mean WM and CSF signals were regressed out using fsl_glm to reduce physiological and scanner-related noise, such as cardiac pulsatility and respiratory fluctuations. This preprocessing pipeline yielded denoised, anatomically aligned, and temporally filtered functional data optimised for subsequent dynamic connectivity analyses (Dipasquale et al., 2017).

### 3.3 Quality control

All structural and functional images were visually inspected for artefacts, motion, and co-registration accuracy at multiple stages of preprocessing. We verified alignment between functional and anatomical images following each registration step, including echo combination, denoising, and MNI normalisation. For the rs-fMRI data, we monitored framewise displacement and excluded any datasets exceeding a mean framewise displacement of 0.5 mm or containing more than 10% of volumes with motion spikes above 0.75 mm. No participants exceeded these thresholds. Independent component classification by ME-ICA was visually examined in a subset of participants to confirm the effective separation of BOLD and non-BOLD components. Additionally, all white matter and CSF masks used for nuisance regression were manually checked for correct anatomical placement in MNI space. These quality control procedures ensured robust and reliable inputs for the dynamic functional connectivity analyses.

### 3.4 LEiDA analyses

We performed Leading Eigenvector Dynamics Analysis (LEiDA) in MATLAB R2020a (MathWorks, Natick, MA, USA) using scripts adapted from Cabral et al. (2017) [https://github.com/juanitacabral/LEiDA]. This method tracks how synchrony between different brain regions evolves over time by analysing the timing of their BOLD signal fluctuations.

**Fig. 1:**
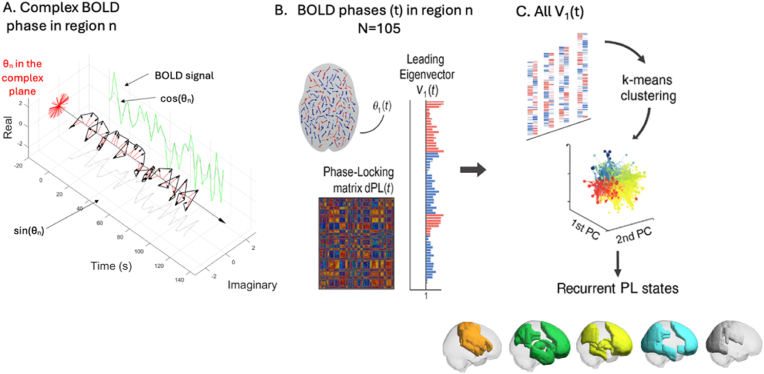
**Schematic overview of the LEiDA pipeline for identifying recurrent brain states.** (A) For each region n, the BOLD signal is bandpass-filtered and transformed into its complex representation to extract the instantaneous phase θn(t) via the Hilbert transform. (B) At each timepoint t, the phases across 105 regions are used to compute a dynamic phase-locking matrix dPL(t), from which the leading eigenvector V1(t) is extracted. V1(t) captures the dominant pattern of phase synchrony across the brain at that time. (C) All leading eigenvectors V1(t) across participants and timepoints are concatenated and submitted to k-means clustering, identifying a set of recurrent phase-locking (PL) states. Each state reflects a spatially distinct and temporally recurring pattern of functional connectivity.

We first extracted BOLD time series from 105 cortical and subcortical regions of interest (ROIs) using the Harvard–Oxford atlas available from the CONN toolbox (Whitfield-Gabrieli and Nieto-Castanon, 2012). Cerebellar ROIs were excluded due to their absence in the Yeo 7-network parcellation. Each ROI time series was then Hilbert-transformed to derive the instantaneous phase in the complex plane (Fig. 1A). At each timepoint, we computed a dynamic phase-locking matrix dPL(t), quantifying pairwise phase synchrony between regions (Fig. 1B). We extracted the leading eigenvector V_1_(t) from each dPL(t) to capture the dominant pattern of whole-brain synchrony at each timepoint. We then concatenated V_1_(t) across all participants and applied k-means clustering to identify recurrent brain states (Fig. 1C). We tested clustering solutions from k = 5 to k = 10 and selected the optimal number of states (k = 5) using the Dunn index, which maximises between-cluster separation and within-cluster homogeneity.

For each identified state, we computed three dynamic metrics per participant: (1) probability of occurrence (proportion of time spent in the state), (2) average lifetime (number of consecutive timepoints within the state), and (3) pairwise transition probabilities between states.

To interpret the functional relevance of each state, we calculated Spearman correlations between the spatial topography of each state's centroid (Vk) and the canonical Yeo 7-network maps (Yeo et al., 2011). These Yeo networks include the visual, somatomotor, dorsal attention, ventral attention, limbic, frontoparietal, and default mode networks. Significance was defined using a Bonferroni-corrected threshold of p < 0.05/7 (0.007). Because large-scale brain networks support multiple cognitive and affective functions, the present interpretations should be viewed as approximations of network-level organization rather than direct evidence of specific psychological states.

### 3.5 Statistical analysis

We compared group differences in LEiDA-derived dynamic metrics using analysis of covariance (ANCOVA), controlling for age, sex, and handedness. For each state, we examined between-group differences in probability of occurrence, lifetime, and transition probabilities. For transition probabilities, all directed pairwise transitions between the five identified states were examined (20 possible transitions excluding self-transitions). Given the exploratory nature of these analyses and the modest sample size, transition probability analyses were not corrected for multiple comparisons and should therefore be interpreted cautiously. For completeness, we supplemented frequentist statistics with their respective Bayesian counterparts to quantify evidence strength. Bayes factors (BF_10_) above 1, 3 and 10 were interpreted as anecdotal, moderate, and strong evidence for a given effect, respectively.

We also performed exploratory correlation analyses between dynamic metrics and behavioural or biological measures, including symptom severity (e.g., fatigue, anxiety), cognitive performance (e.g., sustained attention), and circulating inflammatory markers (e.g., interleukin-6). We used partial Spearman rank correlations, which accounted for age, gender, handedness and group. Correlation analyses between dynamic brain-state metrics and behavioural or biological variables were treated as exploratory and are reported without correction for multiple comparisons.

### 3.6 Sensitivity analyses

To examine the robustness of the main findings, we performed two complementary sensitivity analyses. First, given the significant between-group difference in years of education, we repeated the primary analyses using years of education as a further covariate.

## 4 Results

### 4.1 Sociodemographics and clinical characterisation

Results have been first reported elsewhere (Martins et al., 2026). Here we describe the main findings that might be relevant to interpret the new neuroimaging findings derived from the use of functional BOLD resting-state imaging. The groups were matched for age, sex, ethnicity, and body mass index (BMI), with no significant differences observed across these variables (all p > 0.05; Table 1). However, groups differed significantly in years of education, with HC participants reporting higher educational attainment. PCS participants also had a significantly longer duration since first SARS-CoV-2 infection, and were significantly less likely to have been vaccinated prior to infection. Clinically, individuals with PCS reported significantly greater symptom burden across multiple domains. These included fatigue (both visual analogue scale and multidimensional components), sleep disturbance (PSQI), post-exertional malaise, autonomic dysfunction (COMPASS-31), affective symptoms (HADS depression/anxiety), PTSD-related symptoms, and musculoskeletal complaints (WPI and SS scores). Notably, 40% of PCS participants met criteria for fibromyalgia, compared to none in the HC group. These differences were supported by both frequentist (p < 0.05) and Bayesian inference, with many outcomes yielding strong to extreme evidence in favour of group separation (BF_10_ > 10), particularly for fatigue, post-exertional malaise, and pain sensitivity (Supplementary Table S1).

**Table 1:** **Sociodemographic and clinical characteristics of PCS and healthy control participants.** Group comparisons of demographic and COVID-19-related variables between individuals with Post-COVID-19 Syndrome (PCS) and matched healthy controls (HC). Data are shown as mean (standard deviation) or counts. Between-group differences were assessed using independent samples t-tests or chi-squared tests as appropriate. Bayes factors (BF_10_) quantify evidence for group differences; values > 1 indicate increasing support for the alternative hypothesis. Asterisks (*) denote statistically significant results at p < 0.05 or BF_10_ > 1.

Variable	PCS Mean (std)	HC​ Mean (std)	Statistics	p	B_10_
**Age**	40.6 (10.9)	40.0 (10.4)	T(38) = 0.208	0.837	0.312
**Gender**	18F/2M	17F/3M	χ^2^(1) = 0.229	0.663	N/A
**Ethnicity**	White: 18​ Mixed: 0​ Asian: 1​ Black: 0​ South Asian: 1​ Hispanic: 0	White: 15​ Mixed: 1​ Asian: 1​ Black: 2​ South Asian: 0​ Hispanic: 1​	χ^2^(5) = 5.273	0.384	N/A
**BMI**	23.8 (3.44)	24.4 (3.84)	T(38) = -0.55	0.582	0.346
**SPO_2_ (%)**	99.105 (1.524)	98.842 (1.344)	T(36) = 0.565	0.576	0.357
**Years of education**	17.30 (2.94)	19.35 (3.13)	T(38) = -2.13	0.039​*	1.845
**MRC Dyspnea scale**	0: 45% 1: 45% 2: 10% 3: 0% 4:0%	0: 95% 1: 5% 2: 0% 3: 0% 4: 0%	χ^2^(2) = 11.971	0.003*	N/A
**Time since first infection (months)**	23.0 (9.17)	15.7 (11.5)	T(38) = 2.21	0.033*	0.749
**Vaccination prior infection**	Yes: 4​ No: 16	Yes: 14​ No: 6	χ^2^(1) = 10.101	0.001*	N/A

### 4.2 Clinical routine blood tests

Group comparisons of routine blood parameters revealed no major differences between PCS and HC participants (Supplementary Table S2). However, PCS participants showed numerically higher creatinine and Gamma-GT levels, with corresponding Bayes factors (BF_10_ = 1.232 and 1.735, respectively) providing anecdotal to moderate evidence for group differences. Platelet count also trended higher in the PCS group (p = 0.08; BF_10_ = 1.123). Other metabolic, inflammatory, and hematological markers - including C-reactive protein, white and red cell counts, hemoglobin, and thyroid function - did not differ significantly between groups.

### 4.3 Serum cytokines and glial markers

No statistically significant differences were observed in circulating levels of IFN-γ, IL-1β, IL-6, IL-8, IL-10, IL-13, TNF-α or GFAP (Supplementary Table S3). However, TNF-α (p = 0.116; BF_10_ = 1.094) and S100β (p = 0.149; BF_10_ = 1.025) showed suggestive evidence of elevation in the PCS group, consistent with mild glial or immune activation.

### 4.4 Cognitive performance

Cognitive testing using the Cognitron battery revealed subtle but functionally relevant differences between groups (Supplementary Table S4). PCS participants showed significantly poorer performance on the delayed object memory task (RT_DFE; p = 0.02; BF_10_ = 2.653), as well as trend-level impairments in the Lead Balloon task (abstract reasoning; p = 0.06; BF_10_ = 1.330) and vigilance (Spotter task RT; p = 0.06; BF_10_ = 1.303). Other domains - including motor control, 2D spatial manipulation, and verbal analogies - did not show significant differences. Overall, Bayesian analysis provided moderate evidence for reduced memory and attentional function in PCS.

### 4.5 Identification and characterisation of optimal number of dynamic brain states

**Fig. 2:**
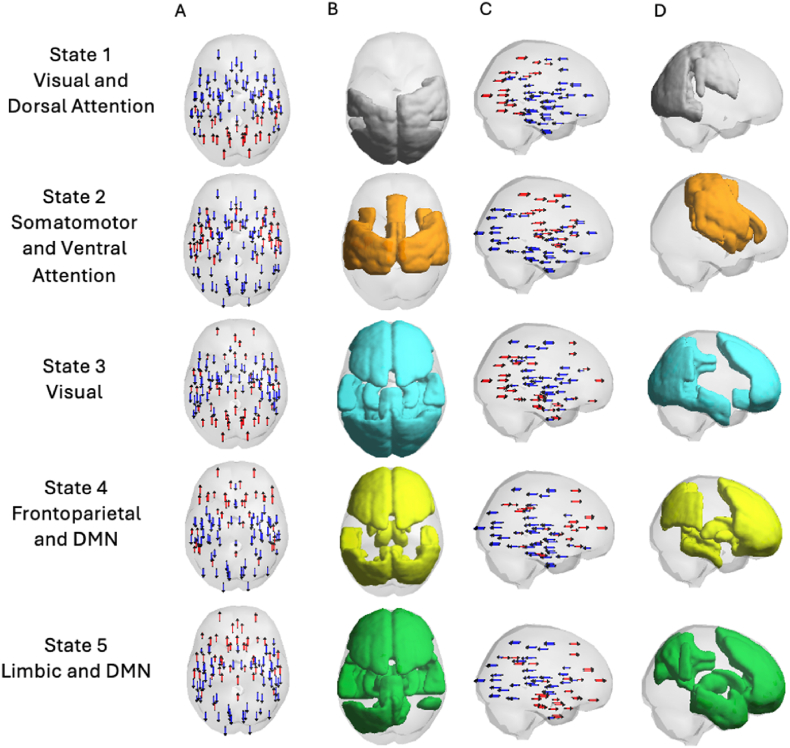
**Recurrent Large-Scale Brain States Identified with LEiDA**. This figure depicts the five dynamic functional brain states extracted using Leading Eigenvector Dynamics Analysis (LEiDA) from resting-state fMRI data. Each row represents one recurrent state, characterized by its dominant spatial organization and network affiliations: (1) Visual and Dorsal Attention, (2) Somatomotor and Ventral Attention, (3) Visual, (4) Frontoparietal and Default Mode Network (DMN), and (5) Limbic and DMN. Column A shows the leading eigenvector phase alignment patterns in axial view, with blue and red arrows indicating opposite phase relationships across regions. Column B highlights the brain areas with the highest contributions to each state, mapped onto inflated cortical surfaces. Column C displays lateral views of the eigenvector vectors to illustrate the directionality and distribution of phase coupling across the cortex. Column D presents an alternative cortical view of the same spatial distribution shown in Column B to aid anatomical interpretation. (For interpretation of the references to colour in this figure legend, the reader is referred to the Web version of this article.)

Using the Dunn index, we identified five recurrent brain states maximising within-state homogeneity and between-state separability. Each state exhibited a distinct spatial configuration of phase synchrony, mapped onto canonical Yeo networks. State 1 primarily involved visual and dorsal attention networks; State 2 showed strong expression in somatomotor and ventral attention regions; State 3 involved mainly visual networks; State 4 was characterised by coactivation of frontoparietal and default mode network (DMN) regions; and State 5 was dominated by limbic and DMN components (Fig. 2 and Table 2).

**Table 2:** **Functional network composition of dynamic brain states.** Each row shows the Spearman correlation (ρ) between the spatial configuration of a given dynamic brain state (States 1–5) and the canonical Yeo 7-network parcellation: Visual, Somatomotor Network (SMN), Dorsal Attention (DA), Ventral Attention (VA), Limbic, Frontoparietal Network (FPN), and Default Mode Network (DMN). Significant correlations after bonferroni correction are marked with an asterisk (*). Values in parentheses indicate uncorrected p-values. State labels reflect the dominant functional networks positively associated with each state.

State number	Labels	Visual	SMN	DA	VA	Limbic	FPN	DMN
1	Visual/DA	0.600* (<0.001)	0.067 (0.494)	0.484* (<0.001)	0.062 (0.530)	−0.359* (<0.001)	0.078 (0.429)	−0.338* (<0.001)
2	SMN/VA	−0.480* (<0.001)	0.610* (<0.001)	0.110 (0.264)	0.529* (<0.001)	−0.304* (0.002)	0.085 (0.388)	−0.328* (<0.001)
3	Visual	0.704* (<0.001)	−0.598* (<0.001)	0.124 (0.208)	−0.540* (<0.001)	0.134 (0.171)	−0.033 (0.735)	0.162 (0.098)
4	FPN/DMN	−0.299* (0.002)	−0.382* (<0.001)	0.315* (0.001)	0.211 (0.030)	−0.013 (0.897)	0.733* (<0.001)	0.442* (<0.001)
5	Limbic/DMN	−0.146 (0.137)	−0.465* (<0.001)	−0.042 (0.672)	−0.294* (0.002)	0.411* (<0.001)	0.242 (0.013)	0.594* (<0.001)

### 4.6 Group differences in brain-state dynamics

**Fig. 3:**
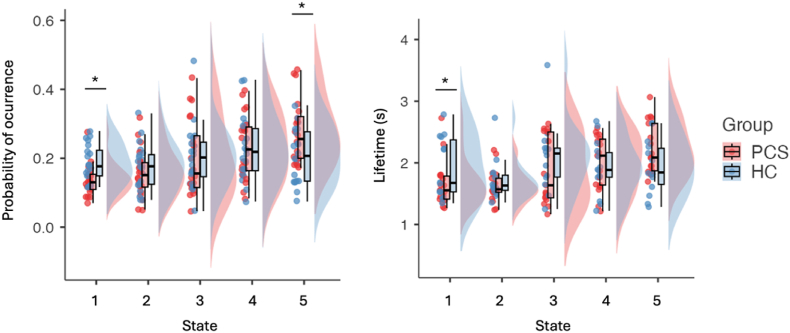
**Group differences in dynamic brain-state metrics between PCS and healthy controls.** Violin plots show (left) the probability of occurrence and (right) the average lifetime (in seconds) for each of the five recurrent phase-locking states identified using LEiDA. Individuals with Post-COVID-19 Syndrome (PCS; red) exhibited a significantly lower probability of occurrence and shorter lifetime in State 1 (visual/dorsal attention) and a significantly higher probability of occurrence in State 5 (limbic/DMN), compared to healthy controls (HC; blue). *p < 0.05, ANCOVA controlling for age, sex, and handedness; error bars represent interquartile range with median. (For interpretation of the references to colour in this figure legend, the reader is referred to the Web version of this article.)

We conducted ANCOVAs controlling for age, sex, and handedness to compare dynamic metrics between individuals with PCS and healthy controls. Participants with PCS exhibited a significantly lower probability of occurrence and shorter lifetime in State 1. Conversely, they showed a higher probability of occurrence for State 5. No significant group differences were found in States 2–4 after correcting for multiple comparisons. Bayesian counterparts generally supported these observations (Table 3 and Fig. 3).

**Table 3:** **Group comparisons of dynamic brain-state metrics between PCS and healthy controls.** Mean (standard deviation) values are shown for the probability of occurrence and average lifetime (in seconds) for each of the five LEiDA-derived brain states. Statistical results reflect ANCOVAs controlling for age, sex, and handedness. *p < 0.05. BF_10_ refers to the Bayes Factor in favour of the alternative hypothesis (PCS ≠ HC), with values > 3 indicating moderate and >10 strong evidence. Significant differences were observed for States 1 and 5 in probability of occurrence, and for State 1 in lifetime.

Metric	State	PCS Mean (SD)	HC Mean (SD)	Statistics	P-value (FDR corrected)	B_10_
**Probability**	1	0.136 (0.045)	0.185 (0.053)	F(1,35) = 9.443	0.004*	13.59*
	2	0.157 (0.069)	0.175 (0.066)	F(1,35) = 0.796	0.378	0.443
	3	0.198 (0.111)	0.205 (0.093)	F(1,35) = 0.045	0.842	0.316
	4	0.233 (0.095)	0.228 (0.0941)	F(1,35) = 0.084	0.774	0.319
	5	0.277 (0.092)	0.207 (0.081)	F(1,35) = 5.862	0.021*	3.57*
**Lifetime**	1	1.66 (0.363)	1.96 (0.466)	F(1,35) = 5.031	0.032*	2.39*
	2	1.67 (0.306)	1.73 (0.318)	F(1,35) = 0.208	0.652	0.359
	3	1.86 (0.553)	2.06 (0.534)	F(1,35) = 1.347	0.254	0.545
	4	2.04 (0.510)	1.96 (0.398)	F(1,35) = 0.389	0.537	0.358
	5	2.23 (0.409)	2.08 (0.620)	F(1,35) = 0.631	0.433	0.417

### 4.7 Transition probabilities

**Fig. 4:**
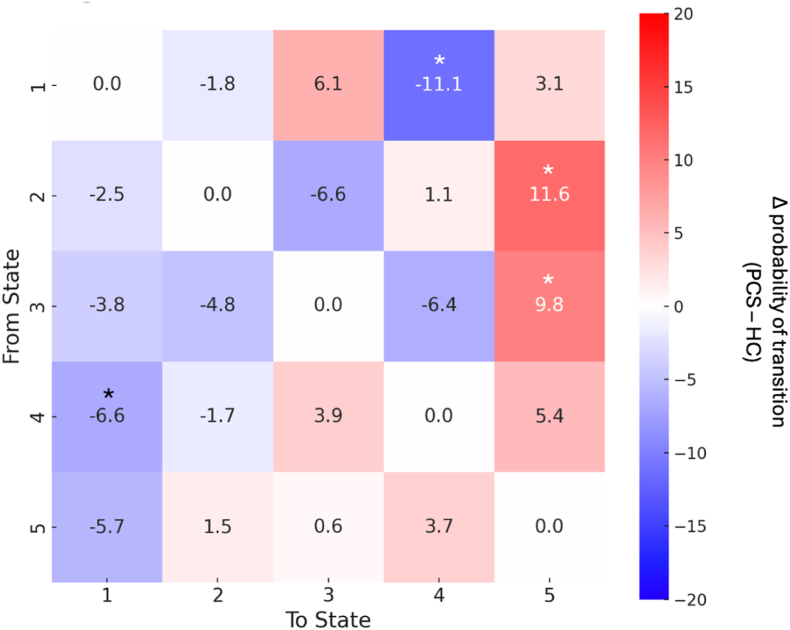
**Group differences in transition probabilities between dynamic brain states.** Heatmap depicts the difference in transition probabilities between PCS and healthy control groups (Δ = PCS − HC) for each pair of phase-locking states. Positive values (red) indicate increased transition probability in PCS; negative values (blue) indicate decreased transition probability. PCS participants showed significantly reduced transitions between State 4 (frontoparietal/DMN) and State 1 (visual/dorsal attention), and significantly increased transitions from State 2 (somatomotor/ventral attention) and State 3 (visual) into State 5 (limbic/DMN). Asterisks denote group differences significant at uncorrected *p < 0.05, ANCOVA controlling for age, sex, and handedness. (For interpretation of the references to colour in this figure legend, the reader is referred to the Web version of this article.)

Exploratory analyses of transition probabilities suggested reduced transitions from State 4 to State 1 and from State 1 to State 4, alongside increased transitions from States 2 and 3 toward State 5 (uncorrected p < 0.05; Fig. 4 and Table 4).

**Table 4:** **Group differences in transition probabilities between dynamic brain states.** Mean transition probabilities (± standard deviation) are shown for all possible pairwise transitions between the five dynamic brain states, separately for the PCS and healthy control (HC) groups. Group comparisons were conducted using ANCOVAs controlling for age, sex, and handedness, with corresponding p-values and Bayes Factors (BF_10_) reported.

	Group	1 → 2	1 → 3	1 → 4	1 → 5	2 → 1	2 → 3	2 → 4	2 → 5	3 → 1	3 → 2	3 → 4	3 → 5	4 → 1	4 → 2	4 → 3	4 → 5	5 → 1	5 → 2	5 → 3	5 → 4
**Mean**	**PCS**	0.193	0.301	0.268	0.277	0.207	0.253	0.257	0.355	0.205	0.218	0.235	0.342	0.177	0.225	0.252	0.356	0.190	0.248	0.239	0.345
	**HC**	0.211	0.240	0.379	0.246	0.232	0.319	0.246	0.239	0.243	0.266	0.259	0.244	0.243	0.242	0.213	0.302	0.247	0.233	0.223	0.308
**SD**	**PCS**	0.105	0.146	0.120	0.128	0.145	0.139	0.168	0.151	0.0828	0.104	0.102	0.124	0.0728	0.0994	0.135	0.164	0.0786	0.104	0.111	0.124
	**HC**	0.124	0.110	0.163	0.129	0.123	0.169	0.101	0.115	0.0915	0.147	0.102	0.134	0.102	0.121	0.120	0.105	0.147	0.105	0.0904	0.119
**Frequentist**	**F(1,32)**	0.452	1.411	7.219	0.553	0.269	1.820	0.122	6.287	1.747	1.499	0.498	5.321	4.969	0.297	1.100	1.359	2.206	0.131	0.362	0.878
	**P-value (FDR-corrected)**	0.506	0.244	0.011	0.462	0.589	0.186	0.729	0.018	0.195	0.229	0.485	0.027	0.032	0.589	0.301	0.252	0.147	0.719	0.551	0.355
**Bayesian**	**B_10_**	0.380	0.616	4.96	0.402	0.354	0.663	0.331	4.11	0.659	0.583	0.382	2.82	2.40	0.350	0.480	0.566	0.770	0.327	0.347	0.435

### 4.8 Sensitivity analyses

Adding years of education as further covariate did not change the main pattern of results with the exception of the significant changes in transitions probability, which should be interpreted with caution.

### 4.9 Exploratory correlations between brain dynamics and clinical, blood and cognitive parameters

We conducted exploratory correlation analyses restricted to dynamic brain-state metrics that showed significant group differences - namely, the probability of occurrence and lifetime of State 1 (visual/dorsal attention) and State 5 (limbic/default mode network). Given the exploratory and hypothesis-generating nature of these analyses, we report uncorrected p-values to facilitate the identification of potential associations for future investigation. The probability of occurrence of State 5 was negatively correlated with global cognitive performance on the MoCA (Spearman's ρ = −0.43, p = 0.015) and with total serum protein levels (ρ = −0.37, p = 0.038), and positively correlated with circulating interleukin-1β (IL-1β; ρ = 0.39, p = 0.027), even though none of these correlations survived FDR correction for the number of parameters tested. In addition, the probability of occurrence of State 1 was positively associated with unrefreshing sleep severity (ρ = 0.34, p = 0.047). However, none of these correlations survived false discovery rate (FDR) correction for multiple comparisons, and should therefore be interpreted with caution (Supplementary Table S5).

## 5 Discussion

This study provides novel evidence that PCS might be associated with altered intrinsic brain dynamics at rest, particularly in large-scale networks supporting attention, sensory integration, and emotion regulation. Using Leading Eigenvector Dynamics Analysis (LEiDA), we identified five recurrent phase-locking states and showed that PCS participants may spend significantly less time in an externally oriented attentional state (State 1: visual/dorsal attention) and more time in an internally affect-focused state (State 5: limbic/default mode network). Exploratory analyses suggested that these alterations were accompanied by reduced transitions between cognitively adaptive states and increased transitions into limbic-dominated configurations. Together, our findings suggest that PCS may involve a shift in baseline brain function away from goal-directed engagement and toward internally driven, potentially affectively charged modes of processing, an imbalance that may offer a fresh brain systems-level perspective on the mechanisms underlying key cognitive and emotional symptoms in PCS. Rather than providing definitive evidence, the present findings delineate a candidate systems-level phenotype of PCS that warrants replication and formal testing in larger, multi-centre cohorts. Given the sample size and dimensionality of the dynamic metrics, these analyses should be interpreted as exploratory.

Our findings resonate with previous studies showing that healthy cognition depends not only on the strength of functional connectivity but also on the dynamic reconfiguration of brain networks over time (Kurtin et al., 2023). In this context, brain-state flexibility - the ability to rapidly shift between large-scale functional configurations - has emerged as a key systems-level property supporting attention, working memory, and emotional control (Kurtin et al., 2023; Cabral et al., 2017). Prior work using LEiDA and other dynamic functional connectivity methods has demonstrated that reduced variability or diminished access to specific dynamic states is associated with clinical conditions marked by fatigue, attentional deficits, and mood disturbances, such as major depression (Alonso Martinez et al., 2020; Alonso et al., 2023; Cahart et al., 2025). Our results suggest that PCS may share this broader signature of dynamic dysregulation, positioning it within a transdiagnostic framework of altered brain-state transitions.

The reduced probability and lifetime in State 1 among PCS participants is particularly compelling. This state overlapped with dorsal attention and visual networks - systems essential for externally directed cognitive control (Vossel et al., 2014; Corbetta and Shulman, 2002). A reduced ability to enter or maintain this state suggests that patients may struggle to sustain attention or shift efficiently between perceptual inputs and task demands (Sadaghiani and D'Esposito, 2015). Importantly, the increased probability of occurrence of State 5, which involved the limbic system and DMN, might indicate an imbalance rather than uniform suppression of dynamics. The limbic-DMN configuration is typically associated with self-referential thought, emotional salience, and interoceptive monitoring (Seeley et al., 2007; Andrews-Hanna et al., 2014). Its overexpression may reflect a pathological bias toward internally focused processing, consistent with reports of emotional dysregulation, rumination, and heightened somatic awareness in PCS (Whitfield-Gabrieli and Ford, 2012; Madden et al., 2025; Scardua-Silva et al., 2024).

Altered transition probabilities in exploratory analyses further reinforce the hypothesis of reduced dynamic repertoire and functional rigidity (Hansen et al., 2015; Vidaurre et al., 2017)**.** In controls, more frequent reciprocal transitions between State 1 and the frontoparietal/DMN state (State 4) may reflect flexible toggling between goal-directed and introspective modes, akin to previously described metastable switching in healthy cognition (Deco et al., 2017; Hellyer et al., 2014). In contrast, PCS participants exhibited attenuated transitions between these states and increased transitions from somatomotor and visual states (States 2 and 3) into the limbic-DMN state (State 5). This asymmetric dynamic trajectory may reflect a form of neural inertia or “gravitational pull” into internally focused, affectively loaded brain modes**,** echoing prior findings in mood disorders and inflammatory syndromes (Zhang et al., 2016; Cocchi et al., 2013; Menon, 2011; Zhou et al., 2020).However, given the exploratory nature of these findings, confirmation in larger and more representative samples is warranted.

While our findings reveal differences in the dynamic organisation of intrinsic brain activity between individuals with PCS and healthy controls, we emphasise the interpretive limitations of resting-state fMRI. The method captures large-scale patterns of synchronised neural activity but does not permit definitive conclusions about causality or mechanism. Interpreting these patterns functionally risks reverse inference, especially given that most cognitive or emotional processes map onto multiple, overlapping neural configurations rather than one-to-one brain-function relationships. By reframing our results through a transdiagnostic lens - where similar alterations in brain-state flexibility have been observed in conditions such as depression - we align with the descriptive strengths of the LEiDA methodology while avoiding speculative or reductive claims. At the same time, we wish to avoid overcompensating in the opposite direction. Emphasising only peripheral or structural explanations risks overlooking more subtle cognitive and emotional contributions to symptom expression - particularly alterations in attention, emotional salience attribution, and interoceptive monitoring. These mechanisms are not mutually exclusive with dysautonomia or inflammation, but may in fact co-express and interact dynamically. Clinically, many individuals with persistent cognitive symptoms following COVID-19 infection present with features closely aligned with functional cognitive disorders - an area still under-recognised by many clinicians and neuroscientists. Contemporary theoretical frameworks, such as those drawing from predictive processing and 'constructed emotion' models, suggest that symptom persistence may reflect impaired updating of internal models about bodily and cognitive states. This can result in 'symptom construction' that continues beyond the resolution of initial peripheral or immune triggers, as seen in other conditions where inflammation-related delirium or fatigue transitions into persistent brain fog.

Although our study was exploratory and not designed to explicitly test such mechanistic frameworks, our findings - particularly the increased expression of internally focused limbic–DMN states and reduced transition flexibility - may be interpreted as systems-level signatures of disrupted adaptive processing. These alterations could reflect a brain that is less responsive to exteroceptive demands and more bound by internally generated expectations or affective salience. Rather than implying that symptoms are “just psychological,” such an interpretation recognises the neurobiological basis of cognitive-emotional processing, the permeability of boundaries between organic and functional symptoms, and the importance of maintaining clinical sensitivity. We believe that adopting a cautious, integrative interpretive stance - grounded in systems neuroscience, informed by clinical experience, and open to multiple explanatory levels - provides the most constructive path forward for research in this area.

Exploratory correlation analyses provided tentative support for the clinical relevance of altered brain-state dynamics in PCS. Specifically, the probability of occurrence of State 5 - a configuration dominated by limbic and default mode network (DMN) regions - was negatively associated with global cognitive performance on the MoCA and positively associated with serum levels of interleukin-1β (IL-1β), a key pro-inflammatory cytokine (Harrison et al., 2009; Marsland et al., 2015). This state may reflect increased engagement of interoceptive and affectively laden neural circuits at the expense of externally directed cognitive functioning. Consistent with neuroimmune models of fatigue and sickness behaviour, sustained peripheral inflammation may bias intrinsic brain dynamics toward bodily monitoring and self-referential processing, potentially impairing cognitive flexibility and engagement with the external environment (Critchley and Harrison, 2013; Dantzer et al., 2008). However, the broader pattern of results warrants caution. More fine-grained analyses using the Cognitron platform, which assesses specific cognitive domains such as attention, working memory, and executive function, did not reveal significant associations with any LEiDA-derived dynamic metrics. Likewise, we found no robust relationships between dynamic state properties and self-reported symptoms, including fatigue and anxiety. These discrepancies suggest that while global measures such as the MoCA may capture diffuse effects of altered brain dynamics, these may not map neatly onto task-specific performance or subjective symptom ratings. It is possible that intrinsic brain dynamics are more closely linked to overall cognitive tone than to discrete cognitive operations or subjective complaints, or that limitations in power and measurement sensitivity may have obscured more subtle associations. However, these correlations did not survive correction for multiple comparisons and should therefore be interpreted cautiously. These results should be viewed as hypothesis-generating and require replication in larger samples.

Our methodology offers both strengths and limitations. The use of multi-echo fMRI with ME-ICA denoising reduces susceptibility to motion and physiological noise, thereby improving signal quality for the analysis of dynamic functional connectivity. The LEiDA framework captures recurring whole-brain patterns of phase synchrony without the constraints of arbitrarily defined sliding windows, and allows for intuitive interpretation of transient brain states. Nonetheless, the modest sample size represents an important limitation. Although comparable to several prior studies examining dynamic functional connectivity using LEiDA, the present findings should be interpreted as preliminary and hypothesis-generating. Replication in larger and more diverse PCS cohorts will be necessary to confirm the stability and generalisability of the observed alterations in brain-state dynamics. Moreover, PCS is a clinically heterogeneous condition, encompassing symptoms such as fatigue, dysautonomia, cognitive impairment, and pain syndromes resembling fibromyalgia. The present study was not powered to examine subgroup differences across these symptom clusters. Future larger studies will be required to determine whether distinct PCS phenotypes are associated with specific alterations in brain-state dynamics. The present study focused on individuals with persistent PCS symptoms following mild acute SARS-CoV-2 infection. This design was chosen to reduce confounding effects related to severe systemic illness or hospitalisation but limits the generalisability of the findings to the broader and clinically heterogeneous PCS population. Future multi-centre studies including patients with varying infection severities, symptom profiles, and disease durations will be necessary to determine whether distinct PCS phenotypes are associated with different alterations in brain network dynamics. The resting-state acquisition duration (∼8 min) provides a reasonable estimate of baseline dynamics, although longer scans may increase sensitivity to rarer or transitional states. Group differences in educational attainment must also be acknowledged: although both groups fell within the high range of educational achievement, controls exhibited significantly higher levels on average. Importantly, none of the brain dynamic metrics correlated significantly with years of education, suggesting that observed group differences are unlikely to be driven by this variable. To further address this potential confound, we conducted sensitivity analyses including years of education as a further covariate and main findings hold. The pattern of results remained consistent, with the same dynamic brain-state metrics showing comparable group differences, indicating that the main findings were robust to differences in educational attainment. Although sensitivity analyses suggested that the principal findings were relatively robust to adjustment for years of education, the groups also differed in time since infection and vaccination status. Because these variables were not jointly modelled in the primary analyses, and because the modest sample size constrained the number of covariates that could be reliably included simultaneously, residual confounding cannot be excluded. Finally, while we anchored our state definitions to the Yeo 7-network atlas to facilitate interpretability, future studies employing finer-grained or individualized parcellations may help further refine the characterization of dynamic brain states. Furthermore, although clustering stability analyses (e.g., split-half or bootstrap resampling) were not performed because the sample sizes were insufficient to allow for unbiased implementation of such analyses, the consistency of state topographies with canonical network organisation and the convergence of effects across independent dynamic metrics support the robustness of the identified states. This poses a limitation in interpreting the generalisability of the current findings. An additional limitation relates to the choice of control group. By comparing PCS participants to individuals who had recovered from SARS-CoV-2 infection, the present design does not allow us to disentangle effects attributable to prior infection per se from those specifically associated with persistent symptomatology. As such, the findings should be interpreted as reflecting differences between *persistent and resolved post-infectious trajectories*, rather than definitive effects of SARS-CoV-2 exposure. Future studies including infection-naïve control groups will be necessary to fully characterise the specificity of these alterations.

In conclusion, our findings provide preliminary evidence that post-COVID-19 syndrome (PCS) may be associated with alterations in the brain's intrinsic dynamic landscape, characterised by reduced engagement of externally oriented attention networks and increased occupancy of internally focused limbic–default mode configurations. This altered balance, together with a potential reduction in the flexibility of transitions between brain states, may be relevant to the fatigue, attentional difficulties, and affective symptoms reported in PCS. However, given the exploratory nature of the study and the modest sample size, these findings should be interpreted with caution. Overall, the results are consistent with a systems-level perspective on PCS and suggest that changes in large-scale brain dynamics may contribute to its clinical presentation. Future studies in larger and independent cohorts will be essential to replicate these findings, clarify their specificity, and determine their potential utility for monitoring or intervention.

## CRediT authorship contribution statement

**Marie-Stephanie Cahart:** Data curation, Formal analysis, Methodology, Software, Writing – review & editing. **Owen O’ Daly:** Methodology, Writing – review & editing. **Ziyuan Cai:** Methodology, Software, Writing – review & editing. **Nicole Mariani:** Data curation, Investigation, Methodology, Writing – review & editing. **Alessandra Borsini:** Investigation, Methodology, Writing – review & editing. **Valeria Mondelli:** Investigation, Methodology, Writing – review & editing. **Brandi Eiff:** Data curation, Investigation, Methodology, Writing – review & editing. **Silvia Rota:** Project administration, Writing – review & editing. **Timothy Nicholson:** Project administration, Writing – review & editing. **Laila Rida:** Methodology, Software, Writing – review & editing. **Adam Hampshire:** Methodology, Software, Writing – review & editing. **Ottavia Dipasquale:** Methodology, Writing – review & editing. **Lia Fernandes:** Writing – review & editing. **Federico Turkheimer:** Resources, Supervision, Writing – review & editing. **Steven C.R. Williams:** Resources, Supervision, Writing – review & editing. **Daniel Martins:** Conceptualization, Data curation, Formal analysis, Funding acquisition, Investigation, Methodology, Project administration, Resources, Supervision, Writing – original draft, Writing – review & editing.

## Declaration of competing interest

The authors declare that they have no known competing financial interests or personal relationships that could have appeared to influence the work reported in this paper.

## Data Availability

Data will be made available on request.
